# The role of structural factors for preventing HIV risk practices among adolescents in South Africa: A three-wave analysis of caregiving, education, food security, and social protection

**DOI:** 10.21203/rs.3.rs-2164051/v1

**Published:** 2023-02-07

**Authors:** William Edward Rudgard, Maria Granvik Saminathen, Boladé Hamed Banougnin, Yulia Shenderovich, Elona Toska

**Affiliations:** University of Oxford; University of Cape Town Centre for Social Science Research; Univeristy of Cape Town Centre for Social Science Research; Cardiff Univeristy School of Social Sciences; University of Cape Town Centre for Social Science Research

**Keywords:** Adolescent, HIV, prevention, condomless sex, transactional sex, structural factors, education, parenting, South Africa

## Abstract

**Background.:**

Structural interventions are endorsed to enhance biomedical and behavioural HIV prevention programmes for adolescents. Aiming to inform future interventions, we evaluated longitudinal associations between six structural factors and five HIV risk practices in a cohort of adolescents in South Africa.

**Methods.:**

We used three rounds of data between 2014–2018 on 1046 adolescents living with HIV and 483 age-matched community peers in South Africa’s Eastern Cape (Observations = 4402). We used multivariable random effects within-between logistic regression to estimate sex-specific associations between six time-varying structural factors − number of social grants, education enrolment, days with enough food, caregiver supervision, positive caregiving, and adolescent-caregiver communication − and five HIV risk practices − multiple sexual partners, transactional sex, age-disparate sex, condomless sex, and sex on substances. We calculated probability differences, contrasting predicted probabilities at average and maximum values of structural factors associated with multiple risk practices.

**Findings.:**

The sample mean age was 15.29 (SD: 3.23) years and 58% were female. In females, compared to average, maximum positive caregiving scores were associated with lower probability of transactional sex (−1.06 percentage points [ppts], 95%CI=−1.60; −0.52ppts), and age-disparate sex (−0.73ppts; 95%CI=−1.26; −0.19ppts); maximum caregiver supervision scores were associated with lower probability of multiple sexual partners (−3.11ppts; 95%CI=−3.87; −2.35ppts) transactional sex (−1.07ppts, 95%CI=−1.42; −0.71ppts), age-disparate sex (−0.67ppts; 95%CI=−1.08; −0.25ppts), condomless sex (−3.96ppts; 95%CI=−5.65; −2.26ppts), and sex on substances (−0.93ppts; 95%CI=−1.50; −0.37ppts); and, seven days with enough food was associated with lower probability of multiple sexual partners (−1.18ppts, 95%CI=−2.06; −0.30ppts), and transactional sex (−0.91ppts; 95%CI=−1.41; −0.42ppts). Relative to non-enrolment, education enrolment was associated with lower probability of age-disparate sex (−3.18ppts; 95%CI=−5.35; −1.01ppts), and condomless sex (−11.32ppts; 95%CI=−19.15; −3.49ppts). In males, compared to average, maximum caregiver supervision scores were associated with lower probability of multiple sexual partners (−2.83ppts; 95%CI=−3.66; −2.00ppts), transactional sex (−0.90ppts; 95%CI=−1.20; −0.60ppts), age-disparate sex (−0.46ppts; 95%CI=−0.77; −0.15ppts), and sex on substances (−1.42ppts; 95%CI=−2.06; −0.78ppts). No other structural factors were associated with multiple risk practices.

**Interpretation.:**

Structural interventions to improve food security and education enrolment among adolescent girls, and positive and supervisory caregiving among adolescent girls and boys are likely to translate into crucial reductions in HIV risk.

## Introduction

In Southern and Eastern Africa, rates of new HIV infections among adolescents and young adults remain well above the Joint United Nations Programme on HIV/AIDS (UNAIDS) elimination targets, at around 220 per 100,000 in 2021 [1, 2]. Within the region, South Africa concentrates 27% of new infections among 15–24 year-olds, despite accounting for only 10% of this population. Adolescent girls and young women face the most severe risk, experiencing incidence rates four times higher than their male peers [3, 4].

Key sexual practices, are known to enhance and perpetuate adolescents’ risk of acquiring or transmitting HIV. Low rates of HIV testing and HIV status disclosure to partners means that condomless sex is a key driver of HIV transmission between sexual partners, especially for vertically infected adolescents [5–8]. Other practices associated with HIV infection include early sexual debut, multiple sexual partners, transactional sex, age-disparate sex, and substance use [9–12]. Acquiring HIV adds a significant burden to adolescence and young adulthood, with challenges related to adherence to antiretroviral therapy, stigma and discrimination, and higher morbidity and mortality [13–15].

Adolescents’ experience of sexual risk practices are shaped by structural factors like poverty, gender inequalities harmful social norms, and access to services [16, 17]. Structural interventions, such as cash transfers, have been endorsed as crucial for enhancing the effects of core HIV prevention programmes like condom distribution, social and behaviour change communication, PrEP, and school based prevention [18]. More recently, there has been growing research into the relative effectiveness of combining different structural interventions for preventing HIV risk (e.g. cash transfers ‘plus’ parenting support) [19]. Several evaluations of ‘combination’ structural interventions are underway, including PEPFAR’s Determined, Resilient, Empowered, AIDS-free, Mentored and Safe (DREAMS) partnership [20–26]. High-quality observational studies should complement these trials by investigating natural levels of structural factors among adolescents and their influence of HIV risk practices.

Aiming to generate timely evidence around effective targets for ‘combination’ structural interventions for HIV prevention among adolescents, we evaluate simultaneous associations between six key structural factors and engagement in five HIV risk practices in South Africa. All analyses are disaggregated by sex, enabling investigation of the distinct influences of structural factors among adolescent girls and boys.

## Methods

The study was a longitudinal analysis using three waves of data from adolescents living with HIV, and community peers, participating in the Mzantsi Wakho (‘Our South Africa’) cohort in the Eastern Cape Province, South Africa [27].

The Eastern Cape is one of the two poorest provinces in South Africa with a Human Development Index of around 0.66, as compared to 0.71 nationally, and 61% of households receiving at least one social grant, as compared to 46% nationally in 2019 [28, 29]. The 2017 South Africa National HIV Prevalence, Incidence, Behaviour and Communication Survey (SABSSM) estimated that 12% of Eastern Cape inhabitants aged 15–24 were living with HIV, and nationally 11% and 5% of female and male 15–24 year-olds were living with HIV, respectively [30].

We report the analysis according to the Strengthening the Reporting of Observational Studies in Epidemiology checklist, Table S1 [31].

### Study sample.

Adolescents and young adults aged 10 to 19 at baseline who had ever initiated HIV care, alongside cohabiting or neighbouring community peers, were recruited in the Amathole District and Buffalo City Metropolitan municipalities of the Eastern Cape between March 2014 and September 2015. All health facilities providing ART to adolescents in the study location were included in the sampling frame (n = 53). In each health facility, paper and electronic clinical les were reviewed to identify adolescents who had ever initiated ART, irrespective of current or past health service attendance. There was 90% uptake at baseline and 93% and 91% survey follow-up between November 2015 and March 2017 (~ 17 months), and between April 2017 and March 2018 (~ 31 months), respectively. An additional 34 adolescents that had initiated HIV care but were not interviewed during the first wave were recruited during the second wave, together with 10 additional community peers.

### Ethics.

Ethical approvals were obtained from the University of Oxford [SSD/CUREC2/12–21; R43892/RE003], University of Cape Town [CSSR 2013/4; CSSR 2019/01], Provincial Departments of Health and Basic Education, health facilities and schools. All adolescents and primary caregivers (where adolescents were under 18 years) gave voluntary informed consent, read aloud in cases of low literacy. No financial incentives were given for participation, but adolescents received a certificate and small gift pack with snacks and toiletries. Interviews took place in Xhosa or English, according to participant choice. Confidentiality was maintained except when participants disclosed serious risk of harm to themselves or others, in which case safeguarding processes were followed. Reports of recent abuse, rape or suicidality were immediately supported with access to post-exposure prophylaxis, pregnancy prevention and child protection measures.

### Measures.

Measures and scales were pre-piloted with a group of local adolescent advisors, and feedback on questionnaire design was given by the South African National Departments of Health, Basic Education, and Social Development, the South African National AIDS Council, UNICEF, PEPFAR, USAID and local NGOs. All questionnaires are available at www.youngcarers.org.za.

#### HIV risk practices.

We assessed five high-risk practices for HIV infection, using questions adapted from the National Survey of HIV and Risk Behaviour amongst young South Africans, the PREPARE trial and the Child Behavior Checklist Youth Self-Report [32, 33]. (1) *Multiple sexual partners*, as reporting more than one sexual partner; (2) *Transactional sex*, as reporting receipt of money, drinks, clothes, mobile airtime, a place to stay, lifts in a car/taxi, better marks at school, school fees, food, or other kinds of material exchange for having sex with someone; (3) *Age disparate sex*, as reporting a sexual partner at least five years older; (4) *Condomless sex*, as reporting not using a condom for the duration of sex at least once; (5) *Sex on substances*, as reporting having sex after drinking or using drugs. All five practices were reported with a recall period of 12 months, except for transactional sex at wave one, which was reported with a recall period of six months.

#### Hypothesised structural factors.

We evaluated six structural factors hypothesised to be associated with lower odds of engaging in risky sexual practices. (1) *Number of social grants*, as the total number of South African Social Security Agency (SASSA) grants received by the adolescent and their household; (2) *Positive caregiving*, as a sum of the six items that make up the positive caregiving subscale of the Alabama Parenting Questionnaire scale, which considers warmth and praise from a primary caregiver (range: 0–24) [34]; (3) *Caregiver supervision*, as a sum of the reverse scores of 10 items that make up the monitoring & supervision subscale of the Alabama Parenting Questionnaire scale, which include setting rules about coming home in evenings, and knowing who an adolescent is friends with (range: 0–40, higher score indicated better supervision) [34]; (4) *Adolescent-Caregiver communication*, as a sum of five items from the Child-Parent Communication Apprehension Scale for use with Young Adults [35]. The scale asks about adolescent-caregiver overall communication as well as communication on sensitive issues, such as medication and sex (range: 0–25); (5) *Education enrolment*, as currently attending school at wave one and two, and currently attending school, university, college, further education or training at wave three; (6) *Days with enough food at home*, as the number of days in the week before the survey with enough food at home. A full summary of questions, and response options relating to HIV risk practices and structural factors is provided in Table S2.

#### Covariates.

We included eight covariates: HIV status at baseline, participant age, rural/urban household location, informal/shack housing, number of household residents (including participant), maternal orphanhood, paternal orphanhood, and an indicator of study wave. Participant HIV status was assessed using clinical les from municipality health facilities.

### Data analysis.

We used four steps in Stata 15. All analyses were disaggregated by sex as there is evidence that structural factors may act differently on HIV risk practices amongst females and males [36]. First, we described sociodemographic characteristics, structural factors, and HIV risk practices overall, by adolescent sex, and by sex, age, and HIV status.

Second, we estimated multivariable associations between our six structural factors and each HIV risk behaviour controlling for key covariates using the random-effects within-between (REWB) modelling framework described in Bell, Fairbrother, and Jones. 2019 [37]. The value of this framework is its decomposition of time-varying predictors into two distinct constituent sources of variation: (i) between-person comparison of individuals’ *average* value of a structural factor over study waves; and (ii) within-person comparisons of individual *deviation* from their average value of a structural factor at each wave. We began by estimating a REWB model (Model 1) including separate within- and between-person effects for time-varying structural factors, and controlling for all eight covariates [37]. The Wald Test was used to compare pairs of within- and between-person coefficients for equality [38]. We then estimated a second model (Model 2) that only included separate within- and between-person effects where there was evidence that they were likely to differ significantly according to the Wald Test (p < 0.05). Throughout our analysis, structural factors were considered as either household-level (number of social grants, positive caregiving, caregiver supervision, and adolescent-caregiver communication), or individual-level (education enrolment and food security). We did not adjust for individual-level factors when investigating household-level structural factors, as they were likely to lie on the causal pathway to study outcomes [39].

Third, for structural factors found to be significantly associated with lower odds of multiple HIV risk practices (either via between-person, within-person, or combined effects) we calculated adjusted probabilities of study outcomes fixing structural factors to ‘0: No’ and ‘1: Yes’ for binary variables and to ‘mean’ and ‘maximum’ for continuous variables. In most cases, maximum scores lay within one standard deviation (SD) of the mean value. Adjusted probabilities were calculated overall, and fixing HIV status to either “0. Not living with HIV” and “1. Living with HIV”. Our prioritisation of structural factors associated with multiple outcomes is motivated by the UNDP ‘accelerator’ concept, which calls for greater focus on approaches to improve multiple outcomes simultaneously [40, 41].

Fourth, as a robustness check for whether variation in structural factors temporally preceded HIV risk practices, we evaluated the association between prior (lagged) structural factors and outcomes measured at the subsequent waves. We used the same approach as for our main analysis, except that among males, we were unable to consider separate within- and between-person effects for education enrolment because of minimal within-person variation.

## Results

The sample included 1563 adolescents, and the total number of observations included in the analysis was 4402, Figure S1. Respondents lost to follow-up data collection were older (p < 0.001) and lived in larger households (p = 0.04) in an urban location (p = 0.03), Table S3. Compared to adolescents living with HIV, community peers at baseline were on average six months older, less likely to be maternally or paternally orphaned, and lived in larger households (all p < 0.001), Table S4. Median time between first and final interview was 951 days. Missing values for all variables were < 10%, except for sex on substances, which was not measured at wave one, Table S5.

### Summary of descriptive characteristics.

Fifty-eight percent of respondents were female and 70% were living with HIV, Table 1. The average age of respondents was 15.29 (SD: 3.23), 26% lived in a rural area, 15% lived in informal housing, 41% were maternally orphaned, 34% were paternally orphaned, and the mean household size was 6.50 (SD: 4.06), Table 1. Compared to males, females were older (p < 0.001), lived in larger households (p = 0.022), and were more likely to live in informal housing (p = 0.003). They were less likely to be enrolled in education (p < 0.001), and on average, reported fewer days with enough food at home last week (p < 0.001). Between- and within-person variability in time-varying structural factors was higher among females, except for within-person variability in caregiver supervision, which was higher among males.

**Figure 1:**
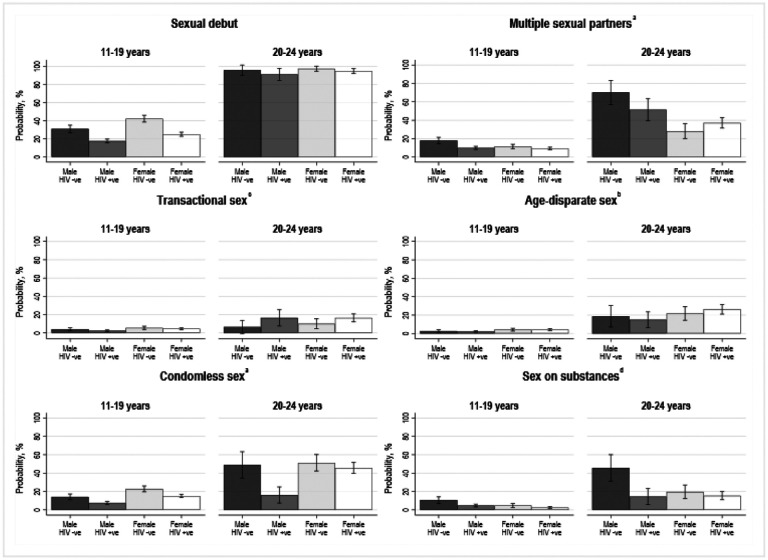
Risk of sexual debut and five HIV risk practices by sex, age, and HIV status over three waves of data collection. N=1563, Observations=4402. ^a^42 observations missing data. ^b^56 observations missing data. ^c^120 observations missing data. ^d^1561 observations missing data upon entry into the study as sex on substances was only measured at waves two and three. Abbreviations: HIV, human immunodeficiency virus.

The two most prevalent HIV risk practices were multiple sexual partners and condomless sex, followed by transactional sex, age-disparate sex, and sex on substances, Table 1. Females were more likely to report transactional sex (p < 0.001), age-disparate sex (p < 0.001), and condomless sex (p < 0.001). Compared to adolescents aged 11–19 years, prevalence of all five practices were significantly higher among young adults aged 20–25 years (p < 0.001), Fig. 1. Compared to their peers not living with HIV, females living with HIV were significantly less likely to report condomless sex (p < 0.001) and sex on substances (p = 0.03), and males living with HIV were less likely to report multiple sexual partners (p < 0.001), condomless sex (p < 0.001), and sex on substances (p < 0.001), Fig. 1. Correlations between study outcomes, and univariable associations between structural factors and outcomes, are summarised in Table S6 and Table S7, respectively.

### Regression analyses.

In models without explanatory variables, values of intra-class correlation (ICC) were > 0.5 for all outcomes − indicating a high correlation between latent HIV risk practices within the same participant across different waves − except for transactional sex in females (ICC = 0.44) and males (ICC = 0.20), age-disparate sex in males (ICC = 0.44), and condomless sex in males (ICC = 0.33), Table S8.

#### Multivariable associations between hypothesised structural factors and HIV risk practices.

In model 1, among females, between- and within-person coefficients differed significantly for caregiver supervision in relation to multiple sexual partners, condomless sex, and sex on substances; education enrolment in relation to transactional sex and condomless sex; and number of days with enough food in relation to transactional sex, Table 2. Among males, between- and within-person coefficients only differed significantly for education enrolment in relation to condomless sex, Table 2.

In model 2, recombining between- and within-person coefficients where there was no evidence of a significant difference, among females, higher positive caregiving scores were associated with lower odds of transactional sex (aOR_combined_=0.94; 95%CI = 0.91, 0.97) and age-disparate sex (aOR_combined_=0.96; 95%CI = 0.92, 0.99); higher caregiver supervision scores were associated with lower odds of multiple sexual partners between-persons (aOR_between_=0.90; 95%CI = 0.86, 0.93), transactional sex (aOR_combined_=0.94; 95%CI = 0.92, 0.96), age disparate sex (aOR_combined_=0.97; 95%CI = 0.94, 0.99), condomless sex between-persons (aOR_between_=0.94; 95%CI = 0.91, 0.97), and sex on substances between-persons (aOR_between_=0.86; 95%CI = 0.80, 0.91); higher adolescent-caregiver communication scores were associated with higher odds of transactional sex (aOR_combined_=1.09; 95%CI = 1.02, 1.16); education enrolment was associated with lower odds of age disparate sex (aOR_combined_=0.44; 95%CI = 0.26, 0.74) and condomless sex between-persons (aOR_between_=0.40; 95%CI = 0.23, 0.69); more days with enough food last week was associated with lower odds of multiple sexual partners (aOR_combined_=0.84; 95%CI = 0.73, 0.96) and transactional sex (aOR_combined_=0.78; 95%CI = 0.68, 0.90), Table 2.

Among males, higher positive caregiving scores were associated with lower odds of transactional sex (aOR_combined_=0.95; 95%CI = 0.90–1.00) and higher odds of condomless sex (aOR_combined_=1.06; 95%CI = 1.02–1.10); higher caregiver supervision scores were associated with lower odds of multiple sexual partners (aOR_combined_=0.93; 95%CI = 0.90, 0.95), transactional sex (aOR_combined_=0.94; 95%CI = 0.91, 0.97), age-disparate sex (aOR_combined_=0.94; 95%CI = 0.91, 0.98), and sex on substances (aOR_combined_=0.92; 95%CI = 0.88, 0.96); education enrolment was associated with higher odds of transactional sex (aOR_combined_=5.56; 95%CI = 1.91, 16.24) and condomless sex within-persons (aOR_within_=2.78; 95%CI = 1.18, 6.58); and days with enough food last week was associated with lower odds of transactional sex (aOR_combined_=0.77; 95%CI = 0.62, 0.96), Table 2. Adjusted odds ratios for the covariates HIV status, rural location, informal housing, household size, maternal orphan, paternal orphan, age, and study wave are summarised in Table S9.

#### Predicted probabilities of HIV risk practices at selected values of ‘accelerator’ structural factors.

**Figure 2:**
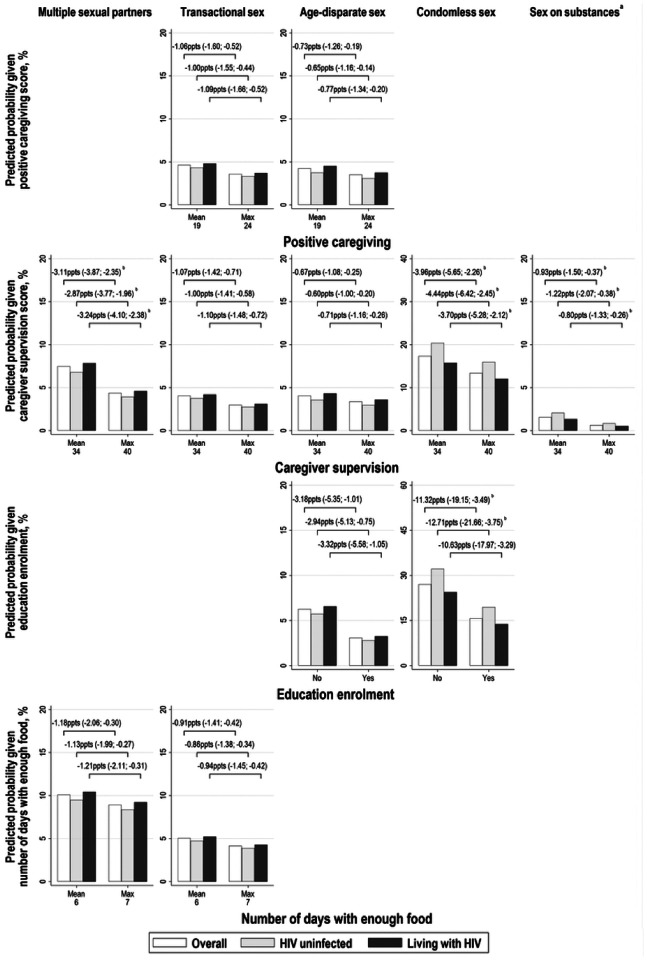
Predicted probabilities of HIV risk practices and probability differences comparing two scenarios among females: (i) in the absence of education enrolment, or at the mean of number of days with enough food, positive caregiving, and caregiver supervision scores; and (ii) in the presence of education enrolment, or at the maximum of number of days with enough food, positive caregiving, and caregiver supervision. Lines connecting bars summarise the calculated difference between two predicted probabilities with 95% confidence intervals in brackets. We only calculated adjusted probabilities where there was evidence of significant associations between structural factors and lower odds of multiple HIV risk practices. Predicted probabilities were also calculated fixing HIV status covariate to “0. Not living with HIV” and “1. Living with HIV”. Values used to build Figure 2 are summarised in Table S11. ^a^Sex on substances was only measured at wave two and wave three. ^b^Predictions are based on between-person effects rather than combined effects. Abbreviations: ppts, percentage points.

**Figure 3:**
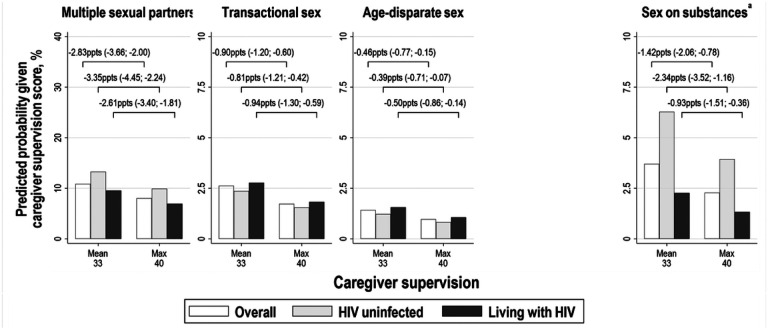
Predicted probabilities of HIV risk practices and probability differences comparing two scenarios among males: (i) at the mean of caregiver supervision; and (ii) at the maximum of caregiver supervision. Lines connecting bars summarise the calculated difference between two predicted probabilities with 95% confidence intervals in brackets. We only calculated predicted probabilities where there was evidence of significant associations between structural factors and lower odds of multiple HIV risk practices. Adjusted probabilities were also calculated fixing HIV status to “0. Not living with HIV” and “1. Living with HIV”. Values used to build Figure 3 are summarised in Table S11. ^a^Sex on substances was only measured at wave two and wave three. Abbreviations: ppts, percentage points.

We summarise predicted probabilities for each of our HIV risk practices at selected values of statistically significant structural factors, and probability differences comparing adjusted probabilities among females in Fig. 2 and males in Fig. 3.

#### Multivariable associations between lagged hypothesised structural factors and HIV risk practices.

Recombining between- and within-person coefficients where there was no evidence of a significant difference, among females, higher prior number of social grants was associated with higher odds of subsequent transactional sex within-persons (aOR_within_=1.43; 95%CI = 1.06, 1.92); higher prior caregiver supervision scores were associated with lower odds of subsequent multiple sexual partners (aOR_combined_=0.94; 95%CI = 0.93, 0.98); transactional sex between-persons (aOR_between_=0.94; 95%CI = 0.89, 0.98), age-disparate sex between-persons (aOR_between_=0.95; 95%CI = 0.90, 0.99), condomless sex (aOR_combined_=0.96; 95%CI = 0.94, 0.98), and sex on substances between-persons (aOR_combined_=0.90; 95%CI = 0.85, 0.96); higher prior number of days with enough food was associated with lower odds of subsequent multiple sexual partners (aOR_combined_=0.81; 95%CI = 0.69, 0.96) and subsequent condomless sex between-persons (aOR_between_=0.82; 95%CI = 0.72, 0.93), Table S10.

Among males, higher prior caregiver supervision and associated with lower odds of subsequent multiple sexual partners between persons (aOR_between_=0.94; 95%CI = 0.89, 0.99), transactional sex between-persons (aOR_between_=0.94; 95%CI = 0.90, 1.00), and sex on substances between-persons (aOR_between_=0.92; 95%CI = 0.86, 0.98). Higher prior caregiver supervision was also associated with higher odds of subsequent transactional sex within-persons (aOR_within_=1.08; 95%CI = 1.02, 1.14), and higher prior adolescent caregiver communication was associated with higher odds of subsequent sex on substances (aOR_combined_=1.14; 95%CI = 1.01, 1.28). Higher prior number of days with enough food was associated with lower odds of subsequent transactional sex within-persons (aOR_within_=0.71; 95%CI = 0.51, 0.97), Table S10.

## Discussion

This study found a high prevalence of five HIV risk practices in a large cohort of adolescents in South Africa. Rates of condomless sex were much higher than the UNAIDS target of no more than 5% for priority groups [42, 43], and young men reported high rates of multiple sexual partners [44]. Compared to their uninfected peers, females living with HIV were significantly less likely to engage in condomless sex, and males living with HIV were less likely to engage in both condomless sex and sex on substances. In gender-stratified analyses, we found that among females, education enrolment, days with enough food at home, positive caregiving, and caregiver supervision were each associated lower probability of multiple HIV risk practices. Among males, caregiver supervision was associated with lower probability of multiple HIV risk practices, and in addition, positive parenting and days with enough food were associated with lower probability of single HIV risk practices. Education enrolment was associated with higher probability of transactional sex and condomless sex, but wide confidence intervals indicate a high level of uncertainty around these estimates. Finally, in lagged analyses we found that in both females and males, prior higher caregiver monitoring and number of days with enough food were associated with lower odds of subsequent HIV risk practices, suggesting that their protective influence may be sustained.

Our analysis applies rigorous statistical methods to three waves of data to investigate relationships between six structural factors and five HIV risk practices simultaneously, further unpacking differential associations for males and females. In females, associations between education enrolment and age-disparate sex and condomless sex, respectively, are consistent with previous analyses and theories supporting this factor’s promotion of safer sexual networks and adolescent girl’s negotiating power [45–50]. The observed association between days with enough food at home and transactional sex is supported by the ‘sex for basic needs’ paradigm of transactional sex [51], and the concurrent relationship between this factor and both multiple sexual partnership and transactional sex is supported by qualitative reports of the influence of poverty on sexual practices among adolescent girls [52]. Associations between caregiver supervision and transactional sex and age-disparate sex are in line with theories suggesting that setting rules and monitoring peer-relationships can act as a ‘protective shield’, promoting the internalization of norms that foster healthy practices, mitigating sensation-seeking and impulsive decision-making, and deterring affiliation with deviant peers [53–56]. Among males, the relationship between caregiver supervision and all five study outcomes can be linked to masculine norms of independence and sexuality driving HIV risk in this population, and caregivers’ gendered perceptions that independence should be encouraged among adolescent boys, while adolescent girls should remain restricted and protected [57, 58].

Our findings support continued emphasis on structural interventions for enhancing the effectiveness of core prevention programmes in HIV prevention [59]. Among females, the range of structural factors associated with HIV risk practices suggests a complex of motivations for engaging in HIV risk practices, and validates the need for comprehensive multi-component prevention approaches in contexts with sufficient resources [19, 60]. Comparing across structural factors, the strong association between education enrolment and age-disparate sex and condomless sex suggests that, in settings with limited resources, interventions should be designed around a core focus to support adolescent girls’ education [61]. There is evidence that such approaches should target both school- and individual-level characteristics, including unaffordable fees [62], poor-quality education and associated learning backlogs [63, 64], early motherhood [65], and low morale for future employment [66]. The finding that caregiver supervision and number of days with enough food may only be associated with small reductions in HIV risk practices indicate that there should be careful consideration of cost-effective approaches for layering multicomponent interventions. Among males, our findings point to a more singular paradigm for engaging in HIV risk practices and a possible role of parenting programmes for encouraging more sustained supervision either by caregivers or other community members such as ‘social fathers’, particularly considering the observed declines in this structural factor by age [67, 68]. Such interventions should be sensitive to local norms around masculinity and transitions to manhood [69]. Finally, findings that HIV risk practices remain high in both adolescents living with HIV and their uninfected peers supports the continued need for population-wide interventions aimed at preventing both new infections and onwards transmission.

This study’s use of three waves of data spanning an average of two and a half years for each participant enabled us to use advanced statistical models to unpack within- and between-person associations between structural factors and HIV risk practices. Nevertheless, there is still a risk of confounding from unmeasured time-varying factors for both between- and within-person associations, and estimated associations should not be interpreted causally. The study’s exhaustive sampling strategy and small loss to follow-up minimises risk of selection bias among participants living with HIV. Although participants not living with HIV were recruited through invitation, the large number of respondents should also minimise selection bias in this group. Self-reported items may be subject to social desirability and recall bias, particularly those relating to sensitive topics [70], and participant subjectivity may also have been a source of measurement error. Because in our study almost all participants reported receipt of at least one social grant (~ 91%) we were unable to robustly evaluate the association between this structural factor and HIV risk practices. Similarly, because so few male participants left education during the study, observed associations between this risk factor and risk practices may not be robust, and should be investigated in future studies.

Building on this study, future mediation analysis could valuably inform the mechanisms via which structural factors act on HIV risk practices and provide stronger causal claim for associations identified in this study. Plausible pathways could include stronger affiliation with a positive peer group, safer sexual networks, and greater negotiating strength. Little research has focused on the influence of peers on sexual practices in South Africa or how peer affiliation is shaped by structural factors [72, 73]. Our finding that even when enrolled in education adolescent girls may still experience a high probability of condomless sex, highlights an urgent needed to identify other structural factors able to address this key sexual practice [74]. Further, evidence indicates that community disorder, caregiver stress, and young men’s social inclusion could be candidates for structural ‘accelerators’ of HIV prevention [75].

## Conclusion

Adolescent girls enrolled in education with greater food security, and adolescent girls and boys experiencing positive and supervisory caregiving are less likely to engage in multiple HIV risk practices. Investments in structural interventions to enhance these factors among young people are likely to translate into crucial progress in reducing HIV incidence.

## Figures and Tables

**Table 1:** Characteristics of participants overall and by sex over three waves of data collection.

	Overall N = 1563 Obs = 4402 n %	Female N = 906 Obs = 2530 n %	Male N = 657 Obs = 1872 n %	p-value^a^
**Sociodemographics**				
Age, mean (SD) [range]	15.29 (3.23) [10–24]	15.78 (3.39) [10–24]	14.63 (2.88) [10–23]	< .001
Living with HIV	3086 (70)	1738 (69)	1348 (72)	0.743
Rural location	1132 (26)	688 (27)	444 (24)	0.088
Informal housing	639 (15)	412 (16)	227 (12)	0.003
Maternal orphan	1796 (41)	983 (39)	813 (43)	0.511
Paternal orphan	1518 (34)	836 (33)	682 (36)	0.053
Household size, mean (SD) [range]	6.39 (3.00) [1–19]	6.51 (3.01) [1–19]	6.22 (2.97) [1–19]	0.022
**Structural factors**				
Number of social grants, mean (SD) [range]	3.16 (2.21) [0–10]	3.23 (2.23) [0–10]	3.07 (2.16) [0–10]	0.833
(Between SD)	(1.88)	(1.92)	(1.83)	
(Within SD)	(1.30)	(1.34)	(1.25)	
Positive caregiving, mean (SD) [range]	18.93 (5.24) [0–24]	18.95 (5.39) [0–24]	18.89 (5.04) [0–24]	0.778
(Between SD)	(3.58)	(3.78)	(3.30)	
(Within SD)	(3.92)	(3.96)	(3.86)	
Caregiver supervision, mean (SD) [range]	33.74 (7.79) [0–40]	33.98 (7.70) [0–40]	33.41 (7.89) [0–40]	0.052
(Between SD)	(5.63)	(5.77)	(5.43)	
(Within SD)	(5.53)	(5.29)	(5.85)	
Adolescent-caregiver communication, mean (SD) [range]	7.24 (2.77) [0–20]	7.21 (2.87) [0–20]	7.28 (2.61) [0–20]	0.220
(Between SD)	(1.81)	(1.91)	(1.68)	
(Within SD)	(2.14)	(2.22)	(2.03)	
Education enrolment	3817 (87)	2040 (84)	1767 (94)	< .001
(Between SD)	(0.30)	(0.36)	(0.19)	
(Within SD)	(0.22)	(0.25)	(0.17)	
Days with enough food at home last week, mean (SD) [range]	6.53 (1.07) [0–7]	6.46 (1.14) [0–7]	6.62 (0.97) [0–7]	< .001
(Between SD)	(0.76)	(0.82)	(0.67)	
(Within SD)	(0.77)	(0.81)	(0.70)	
**Sexual practices**				
Sexual debut	1516 (34)	1035 (41)	481 (26)	< .001
Multiple sexual partners^b^	625 (14)	346 (14)	279 (15)	0.481
Transactional sex^c^	224 (5)	161 (6)	63 (3)	< .001
Age-disparate sex^d^	238 (5)	182 (7)	56 (3)	< .001
Condomless sex^b^	758 (17)	561 (22)	197 (11)	< .001
Sex on substances^e^	202 (5)	103 (4)	99 (5)	0.156

1519 participants were first interviewed at wave one of data collection, and 44 participants were first interviewed at wave two of data collection.

a Estimated from univariable random effects regression to account for clustered nature of data.

b 42 observations missing data.

c 56 observations missing data.

d 120 observations missing data.

e 1561 observations missing data upon entry into the study as sex on substances was only measured at waves two and three.

Abbreviations: Obs, observations; HIV, human immunodeficiency virus; SD, standard deviation.

**Table 2:** Multivariable associations between hypothesised structural factors and HIV risk practices in female and males. N = 1563, Observations = 4402.


	Multiple sexual partners			Transactional sex			Age-disparate sex			Condomless sex			Sex on substances^a^		
	Model 1		Model 2	Model 1		Model 2	Model 1		Model 2	Model 1		Model 2	Model 1		Model 2
	aOR (95%CI)	p^c^	aOR (95%CI); p^d^	aOR (95%CI)	p^c^	aOR (95%CI); p^d^	aOR (95%CI)	p^c^	aOR (95%CI); p^d^	aOR (95%CI)	p^c^	aOR (95%CI); p^d^	aOR (95%CI)	p^c^	aOR (95%CI); p^d^
**Female**															
**Household-level factors** ^b^															
**Number of social grants**															
Between	1.00 (0.85–1.18)	0.915		0.98 (0.82–1.17)	0.952		0.96 (0.79–1.16)	0.129		1.10 (0.96–1.24)	0.862		0.95 (0.74–1.23)	0.174	
Within	0.99 (0.86–1.14)			0.99 (0.83–1.18)			1.15 (0.96–1.37)			1.08 (0.97–1.21)			0.75 (0.56–1.01)		
Combined			0.99 (0.88–1.12); 0.871			0.99 (0.86–1.14); 0.875			1.06 (0.91–1.22); 0.466			1.09 (0.99–1.20); 0.070			0.87 (0.70–1.07); 0.189
**Positive caregiving**															
Between	1.01 (0.95–1.07)	0.237		0.93 (0.88–0.98)	0.628		0.95 (0.89–1.01)	0.632		1.01 (0.96–1.05)	0.575		1.03 (0.96–1.11)	0.732	
Within	0.97 (0.93–1.00)			0.95 (0.90–0.99)			0.96 (0.92–1.01)			0.99 (0.96–1.02)			1.05 (0.97–1.14)		
Combined			0.98 (0.95–1.01); 0.179			**0.94 (0.91–0.97); <.001**			**0.96 (0.92–0.99); 0.017**			1.00 (0.97–1.02); 0.718			1.03 (0.98–1.09); 0.266
**Caregiver supervision**															
Between	0.90 (0.86–0.93)	**< .001**	**0.90 (0.86–0.93); <.001**	0.92 (0.89–0.96)	0.224		0.94 (0.90–0.98)	0.139		0.94 (0.91–0.97)	**< .001**	**0.94 (0.91–0.97); <.001**	0.86 (0.81–0.91)	**0.001**	**0.86 (0.80–0.91); <.001**
Within	1.02 (0.99–1.04)		1.02 (0.99–1.04); 0.170	0.95 (0.93–0.98)			0.98 (0.95–1.01)			1.01 (0.99–1.03)		1.01 (0.99–1.03); 0.427	0.98 (0.93–1.02)		0.96 (0.92–1.00); 0.058
Combined						**0.94 (0.92–0.96); <.001**			**0.97 (0.94–0.99); 0.005**						
**Adolescent-caregiver communication**															
Between	0.94 (0.85–1.05)	0.378		1.11 (0.99–1.24)	0.662		0.93 (0.83–1.05)	0.343		1.01 (0.93–1.09)	0.437		0.95 (0.82–1.10)	0.601	
Within	1.00 (0.94–1.07)			1.08 (0.99–1.17)			1.00 (0.92–1.08)			0.97 (0.92–1.02)			0.90 (0.78–1.03)		
Combined			0.98 (0.93–1.04); 0.543			**1.09 (1.02–1.16); 0.014**			0.98 (0.92–1.04); 0.507			0.98 (0.94–1.02); 0.401			0.93 (0.84–1.02); 0.136
**Variance components**															
Intercept	2.52		2.52	1.10		1.11	2.10		2.05	1.59		1.59	3.43		3.16
**Goodness of fit**															
AUC	0.96		0.96	0.93		0.93	0.96		0.96	0.93		0.93	0.99		0.98
**Individual-level factors**															
**Education enrolment**															
Between	1.34 (0.76–2.37)	0.536		1.48 (0.85–2.58)	0.293		0.40 (0.21–0.77)	0.241		0.36 (0.23–0.56)	**< .001**	**0.40 (0.23–0.69); <.001**	0.79 (0.31–1.97)	0.940	
Within	1.70 (1.05–2.76)			2.29 (1.21–4.30)			0.69 (0.37–1.31)			1.23 (0.81–1.87)		0.94 (0.57–1.54); 0.797	0.83 (0.26–2.68)		
Combined			1.07 (0.68–1.68); 0.763			1.45 (0.85–2.46); 0.168			**0.44 (0.26–0.74); 0.002**						0.81 (0.39–1.68); 0.567
**Days with three meals**															
Between	0.90 (0.73–1.10)	0.902		0.75 (0.62–0.90)	0.479		1.09 (0.85–1.39)	0.741		0.87 (0.75–1.02)	0.067		0.98 (0.67–1.45)	0.648	
Within	0.91 (0.79–1.05)			0.82 (0.70–0.96)			1.14 (0.93–1.40)			1.04 (0.93–1.17)		1.10 (0.96–1.26); 0.186	0.87 (0.60–1.25)		
Combined			**0.84 (0.73–0.96); 0.009**			**0.78 (0.68–0.90); <.001**			1.15 (0.95–1.38); 0.145						0.92 (0.71–1.19); 0.523
**Variance components**															
Intercept	2.68		2.61	0.69		1.15	2.04		2.13	1.33		1.53	3.32		3.33
**Goodness of fit**															
AUC	0.96		0.96	0.94		0.94	0.96		0.96	0.93		0.93	0.98		0.99
**Male**															
**Household factors** ^b^															
**Number of social grants**															
Between	1.06 (0.87–1.29)	0.939		1.04 (0.84–1.29)	0.878		0.98 (0.75–1.28)	0.719		1.07 (0.92–1.23)	0.762		1.02 (0.80–1.30)	0.681	
Within	1.05 (0.89–1.23)			1.02 (0.81–1.28)			0.92 (0.70–1.20)			1.10 (0.95–1.27)			1.10 (0.84–1.43)		
Combined			1.05 (0.92–1.20); 0.449			1.03 (0.87–1.22); 0.697			0.95 (0.77–1.16); 0.623			1.08 (0.97–1.21); 0.141			1.05 (0.87–1.27); 0.602
**Positive caregiving**															
Between	1.02 (0.93–1.11)	0.752		0.99 (0.90–1.08)	0.258		1.03 (0.91–1.16)	0.711		1.05 (0.99–1.12)	0.626		1.06 (0.96–1.18)	0.848	
Within	1.00 (0.95–1.05)			0.92 (0.86–0.99)			1.00 (0.93–1.08)			1.07 (1.02–1.12)			1.05 (0.97–1.14)		
Combined			1.00 (0.96–1.05); 0.854			**0.95 (0.90–1.00); 0.041**			1.01 (0.95–1.08); 0.759			**1.06 (1.02–1.10); 0.001**			1.05 (0.98–1.12); 0.137
**Caregiver supervision**															
Between	0.91 (0.87–0.96)	0.439		0.92 (0.88–0.97)	0.392		0.94 (0.88–1.00)	0.882		0.97 (0.94–1.01)	0.344		0.89 (0.83–0.95)	0.225	
Within	0.93 (0.91–0.96)			0.95 (0.91–0.99)			0.95 (0.90–0.99)			1.00 (0.97–1.02)			0.94 (0.89–0.99)		
Combined			**0.93 (0.90–0.95); <.001**			**0.94 (0.91–0.97); <.001**			**0.94 (0.91–0.98); 0.003**			0.99 (0.97–1.01); 0.253			**0.92 (0.88–0.96); <.001**
**Adolescent-caregiver communication**															
Between	1.04 (0.91–1.20)	0.987		1.06 (0.92–1.22)	0.620		0.90 (0.74–1.10)	0.348		0.96 (0.87–1.06)	0.998		0.98 (0.83–1.16)	0.590	
Within	1.04 (0.96–1.13)			1.01 (0.90–1.14)			1.01 (0.88–1.15)			0.96 (0.89–1.04)			0.92 (0.79–1.07)		
Combined			1.04 (0.97–1.12); 0.277			1.04 (0.95–1.14); 0.430			0.98 (0.88–1.09); 0.661			0.96 (0.90–1.02); 0.193			0.95 (0.85–1.06); 0.353
**Variance components**															
Intercept	2.67		2.66	0.00		0.00	1.68		1.71	0.60		0.63	3.36		3.26
**Goodness of fit**															
AUC	0.97		0.97	0.86		0.86	0.96		0.96	0.87		0.88	0.98		0.98
**Individual factors**															
**Education enrolment**															
Between	1.09 (0.32–3.76)	0.395		6.24 (1.45–26.89)	0.852		1.85 (0.33–10.30)	0.784		0.68 (0.27–1.73)	**0.029**	0.69 (0.28–1.75); 0.438	2.46 (0.68–8.82)	0.126	
Within	2.12 (0.85–5.29)			5.24 (1.32–20.78)			1.34 (0.34–5.22)			2.78 (1.18–6.59)		**2.78 (1.18–6.58); 0.020**	0.52 (0.12–2.26)		
Combined			1.68 (0.80–3.53); 0.168			**5.56 (1.91–16.24); 0.002**			1.52 (0.56–4.12); 0.410						1.27 (0.50–3.22); 0.619
**Days with three meals**															
Within	1.03 (0.71–1.50)	0.987		0.71 (0.51–0.98)	0.511		1.01 (0.61–1.67)	0.790		1.11 (0.84–1.47)	0.264		1.08 (0.68–1.70)	0.849	
Between	1.03 (0.78–1.35)			0.83 (0.60–1.15)			1.10 (0.69–1.76)			0.90 (0.70–1.15)			1.01 (0.63–1.63)		
Combined			1.03 (0.82–1.29); 0.823			**0.77 (0.62–0.96); 0.019**			1.06 (0.75–1.51); 0.738			0.99 (0.82–1.18); 0.897			1.05 (0.76–1.46); 0.766
**Variance components**															
Intercept	2.69		2.69	0.00		0.00	1.75		1.70	0.66		0.66	3.27		3.18
**Goodness of fit**															
AUC	0.97		0.97	0.87		0.85	0.96		0.96	0.88		0.88	0.98		0.98

Coefficients for additional covariates are available in Table S7.

a Sex on substances was only measured at wave two and wave three.

b We did not adjust for education enrolment and days with three meals as we expected them to lie on the causal pathway between receipt of social grants and study outcomes.

c Wald p-value for equality across pairs of within-person and between-person coefficients.

A significant p-value indicates that pairs of coefficients are different from one another.

d Wald p-value for significance.

A significant p-value indicates that a coefficient is significantly different from 1.00. Abbreviations: aOR, adjusted odds ratio; CI, confidence interval; AUC, Area Under Curve.

## Data Availability

The data that support the findings of this study are available from the corresponding author upon reasonable request.
